# Impact of growing up with somatic long-term health challenges on school completion, NEET status and disability pension: a population-based longitudinal study

**DOI:** 10.1186/s12889-021-10538-w

**Published:** 2021-03-16

**Authors:** Anurajee Rasalingam, Idunn Brekke, Espen Dahl, Sølvi Helseth

**Affiliations:** 1 Faculty of Health Sciences, Department of Nursing and Health Promotion, Oslo Metropolitan University, Oslo, Norway; 2 Faculty of Health Sciences, Department of Nursing and Health Promotion, Oslo Metropolitan University, Oslo, Norway; 3 Divison of Child Health and Development, Norwegian Institute of Public Health, Oslo, Norway; 4 Faculty of Social Sciences, Department of Social Work, Child Welfare and Social Policy, Oslo Metropolitan University, Oslo, Norway

**Keywords:** Longitudinal study, Young adults, Adolescents, Long-term illness, Chronic condition, Transition to adulthood, Educational outcomes, Vocational outcomes

## Abstract

**Background:**

Young adulthood is an important transitional life phase that can determine a person’s educational and employment trajectories. The aim of this study was to examine the impact of somatic long-term health challenges in adolescence on upper secondary school completion, not in education, employment or training (NEET status) and receiving disability pension in early adulthood. Additional disparities in educational and employment achievements were also investigated in relation to socioeconomic background.

**Methods:**

The sample consisted of all young adults born in the period 1990 to 1996, (*N* = 421,110). Data were obtained from the Norwegian Patient Registry which is linked to the Central Population Register, education and income registries and the Historical Event Database in Statistics Norway. These data sources provide longitudinal population data. Statistical analyses were performed using multiple logistic regression and computed average marginal effects after the multiple logistic regression.

**Results:**

The results showed that, compared to young adults without long-term health challenges, young adults with the diagnoses inflammatory bowel disease, epilepsy, diabetes, sensory impairment, spinal muscular atrophy (SMA), spina bifida (SB) and cerebral palsy (CP) had lower odds of completing upper secondary education. Moreover, young adults with long-term health challenges had higher odds of NEET status by age 21 compared to those without a long-term health challenge. As for the odds of NEET status by age 21, the results showed that young adults with epilepsy, SMA, SB and CP in particular had the highest odds of receiving disability pension compared to young adults without long-term health challenges.

**Conclusions:**

This longitudinal study revealed that on average young adults with long-term health challenges, compared to those without, struggle to participate in education and employment. The findings highlight the need for preventive measures especially in relation to young adults with neurological conditions such as epilepsy, SMA, SB, and CP.

## Background

The prevalence of chronic conditions and disability (long-term health challenges) in childhood and adolescence is increasing throughout the world [1], and it is estimated that approximately 10–15% of young people live with a long-term health challenge. The prevalence is influenced by the diversity in demographics, definitions and methodology used [2]. Somatic long-term health challenges relate to health problems that require ongoing management over a period of years or decades [3, 4]. Depending on the severity of the long-term health challenge, this may require hospitalizations and extensive medical care which influence adolescents’ everyday lives in various ways [5, 6] and may have severe consequences for their wellbeing [7].

The aim of this article is to shed light on the educational and employment outcomes in early adulthood of growing up with long-term health challenges during adolescence. Participation in education and employment is considered an important factor in the transition to promote adult quality of life [8]. A number of adolescents growing up with long-term health challenges experience problems at school [9] and as a result may not complete upper secondary school with formal qualifications [2]. Furthermore, previous research has shown that long-term health challenges in childhood and adolescence have an adverse effect on immediate and longer-term employment [10–12] which is exacerbated by low educational attainment [13–15]. Young people who are not in education, employment or training (NEET) have a higher risk of several long-term personal and societal economic consequences [16–18]. The term NEET covers unemployed and inactive young people not enrolled in any formal or non-formal education. The NEET concept has proved a powerful tool in enhancing understanding of young people’s vulnerabilities in terms of labour market participation and social inclusion [19]. In Norway, 9.7% of young people aged 15–29 were registered as NEETs in 2016, whereas the average proportion of NEETs in OECD countries was 15.3% [20]. Not completing upper secondary education may to a large extent explain a forthcoming NEET status and has proven to be an independent risk factor for future use of welfare benefits [21, 22]. Having a long-term health challenge and being dependent on medical or vocational rehabilitation indicates work marginalization and a risk of long-term sick leave [23, 24], which is a predictor of disability pension [25]. In Norway, disability pension provides a secure income to those who have a permanently reduced earning capacity due to illness, injury or disability. To be entitled to disability pension the applicant must be between 18 and 67 years old, have been a member of the national insurance program for at least 3 years (all residents of the country are members), and undergone appropriate medical treatment and rehabilitation that might improve their ability to work [26, 27]. The number of young people aged 18–29 receiving disability pension in Norway has increased significantly during the last 10 years [28]. Nearly half of Norwegian NEETs receive health-related benefits, and one in five are still in the same situation 5 years later [29]. Given the heterogeneity of the NEET population in Europe, more knowledge is needed about young people who are at risk of early work disability in order to ensure policy measures target specific subgroups [30].

Hale, Bevilacqua [31] found in their systematic review that there was a clear association between various mental health conditions in adolescence and poor educational outcomes and unemployment. However, the evidence base for physical health conditions was less available and mixed findings emerged. Existing research in this field focusing on physical disability is also limited by the use of cross-sectional design. To address this knowledge gap, the purpose of this study is to examine the impact of physical long-term health challenges, with respect to discrepancies between different types of physical long-term health challenges on a young person’s upper secondary school completion, NEET status and receiving disability pension in a population-based longitudinal study. Previous research has documented associations between parental educational attainment and their children’s ability to perform in education and integrate into the labour market in adult life [32]. We therefore also aim to investigate the influence of parental education on these outcomes among young adults within the specific types of long-term health challenges compared to those of young adults without long-term health challenges.

## Methods

### Design and sample

The sample in the present study consists of all young adults born in the period 1990 to 1996, living in Norway at the age of 21 (*N* = 421,110). Due to data availability, the outcomes were observed for the cohorts born in 1990–1996. The outcome variables were measured when the youth turn 21 and are collected from the time period 2011 to 2017 (i.e. youth born in 1990 turn 21 in 2011, and youth born in 1996 turn 21 in 2017). The data used in this study originate from several public registers in Statistics Norway. Information on adolescents with long-term health challenges is taken from the Norwegian Patient Registry (NPR) in the period 2008 to 2017. Information on variables such as country of origin, gender, age, labour market outcomes, income and welfare benefits (disability pension) are taken from the Historical Event Database, FD-Trygd and were available for the period 2006 to 2017. Information on education is taken from the National Education Database (NUDB) and were available for the same time period as the FD-Trygd data. These data sources provide longitudinal population data linking parents and young adults. The study was approved by the Norwegian South-East Regional Committee for Medical and Health Research (2017/1297).

### Data

#### Outcome variables

The three dependent variables for the study are binary response data. The first dependent variable indicates whether the young adult has completed upper secondary education by age 21 (yes = 1, no = 0). The second variable measures whether the young adult is registered as not in employment, education or training (NEET) by age 21 (yes = 1, no = 0). This variable is based on the following two variables, “in education” and “employment status”. The variable “in education” is coded “1” if they were registered in the educational system by age 21 and “0” if not. The variable “employment status” is measured in the reference week in October and is coded as follows: 0) not employed, 1) employed, 2) employee, 3) unemployed, 4) in training. This variable was then dichotomised; employed, employee, and in training were coded as “1” and not employed and unemployed as “0”. The youth are classified in relation to their situation in that reference week. The third dependent variable indicates whether the young adult has received disability pension by age 21 (yes = 1, no = 0).

#### Exposure variables - long-term health challenges

The NPR is an administrative database of records reported by all government-owned hospitals and outpatient clinics, and by all private health clinics that receive government assistance. The registry contains individual information about all treatment provided by the specialist healthcare service. We identified adolescents with long-term health challenges using the NPR Diagnostic codes, which are based on the World Health Organization’s International Classification of Diseases, version 10 (ICD-10). From 2008, NPR could be linked to other national registers. The following diagnoses were measured using (ICD-10) diagnostic codes: 1) celiac disease (k90.0); 2) asthma (J45,J46); 3) inflammatory bowel disease (IBD) (K50,K51); 4) epilepsy (G40,G41); 5) diabetes (E10); 6) juvenile arthritis (M08); 7) sensory impairments (H54, H90) and 8) spinal muscular atrophy (SMA), spina bifida (SB) and cerebral palsy (CP) (G12,Q05,G80). Due to the low number of recorded diagnoses of spina bifida and spinal muscular atrophy, the diagnosis groups were merged into one group together with CP because they are similar in terms of impaired physical abilities, as done in other studies [33]. These selected diagnoses are some of the most common somatic long-term health challenges among adolescents [5, 6] (see appendix for supplementary information regarding each diagnosis). We have distinguished between these long-term health challenges because they may differ in terms of severity and ability to perform in education and work. Young people not registered in the NPR in the observation period from 2008 until 2017 were considered the reference population.

#### Confounders

Gender was categorised as: girls =1 and boys = 0. Immigrant background was categorised as “majority population” which means born in Norway with at least one Norwegian-born parent, “first-generation immigrants” and “second-generation immigrants”. Parental socioeconomic status (SES) was measured by separate variables for parental education and parental income. Parental education constituted the education level of the parent with the highest education or of the only parent who was present. Parental education was divided into two levels and coded as 0 for upper secondary education or below, or 1 for any college/university education. Parental income was measured as both parents’ gross combined mean annual income from the year the young people turned 16 years old. Income included salary, income from self-employment and state support benefits, e.g. unemployment, sickness and maternity. The logarithm of parental income was used in the analyses. Birth cohort is also adjusted for in the analysis and ranges from 1990 to 1996. Finally, we also control for whether the young people had been treated for any mental health problems in the specialist healthcare service registered in NPR.

#### Analysis

Descriptive analyses are presented with means (SD) and proportions (%). Multiple logistic regression analyses were performed to analyse the impact of long-term health challenges on the odds of school completion, NEET status and receiving disability pension by age 21. Because the coefficients in logistic regression not only reflect the effects of the independent variables but also the size of the unobserved heterogeneity [34], we have computed average marginal effects (AME) after the multiple logistic regression. The marginal effects for the categorical variables indicate how P (Y = 1) (P = probability, Y = outcome variable) changes as the categorical independent variable changes from 0 to 1, holding all other independent variables equal. For continuous independent variables, the marginal effect measures the instantaneous rate of change [35]. To estimate whether the impact of parental education on the outcome variables is dependent on the type of long-term health challenges, we have included an interaction term between parental education and long-term health challenges. We used “pwcompare” after the multiple logistic regression to obtain differences within the diagnosis groups. Statistical analysis was performed using STATA® 16, computing AMEs with the margins command. The statistical significance level was set to *p* < 0.05.

## Results

### Descriptives

Table 1 provides sample characteristics. In total, 8.17% of the sample grew up with a long-term health challenge. Most adolescents with a long-term health challenge had asthma (3.30%) or sensory impairment (1.25%). Approximately 11% of the participants in our sample have an immigrant background. Among first-generation immigrants with long-term health challenges, most adolescents had sensory impairment (5.80%) or SMA, SB and CP (6.02%) and among second-generation immigrants with long-term health challenges most adolescents had inflammatory bowel disease (4.57%) and SMA, SB and CP (4.44%). Overall, more adolescents had parents with upper secondary education and below than college or university education. Adolescents with epilepsy had received most mental health care treatment (13.48%).

**Table 1:** Characteristics of the study population, presented as proportion

*N* = 421,110	Reference population	Celiac disease	Asthma	IBD	Epilepsy	Diabetes	Juvenile arthritis	Sensory impairment	SMA / SB /CP
Number of adolescents (%)	386,690 (91.83)	3453 (0.82)	13,881 (3.30)	1882 (0.45)	4569 (1.08)	3118 (0.74)	998 (0.24)	5257 (1.25)	1262 (0.30)
Girls (%)	48.06	69.50	50.97	52.44	49.44	44.00	65.63	50.62	46.67
Majority (%)	89.69	95.66	94.26	92.40	91.70	95.38	96.29	91.10	89.54
1. generation (%)	6.38	1.97	2.38	3.03	4.92	2.47	2.10	5.80	6.02
2. generation (%)	3.93	2.37	3.36	4.57	3.37	2.15	1.60	3.10	4.44
Parental education: upper secondary education and below (%)	52.92	49.52	52.06	55.59	56.42	56.70	52.30	56.71	55.47
Parental education: any college/university education (%)	44.90	49.81	47.17	42.08	41.72	42.30	47.49	41.15	42.87
Parental income (log income), mean and (SD)	12.6 (0.67)	12.7 (0.49)	12.7 (0.44)	12.7 (0.38)	12.6 (0.62)	12.6 (0.58)	12.7 (0.35)	12.6 (0.65)	12.6 (0.54)
Treated in mental health care (%)		5.47	6.51	7.70	13.48	9.01	7.01	8.33	7.69

Note: parental education unknown: reference population (2.18%), celiac disease (0.67%), asthma (0.77%),  IBD (1.33%), epilepsy (1.86%), diabetes (0.99%), juvenile arthritis (0.20%), sensory impairment (2.15%), SMA/ SB/CP (1.66%). Parental income: presented as mean (SD) of annual income in thousands

### Upper secondary school completion, NEET status and receiving disability pension by age 21

Estimates from the logistic regression of the odds of upper secondary school completion, NEET status and receiving disability pension by age 21 are shown in Tables 2, 3 and 4 as ORs and AMEs. The results in Table 2 show that the odds of completing upper secondary education were significantly lower for young adults with IBD, epilepsy, diabetes, sensory impairment and young adults with SMA, SB and CP compared to healthy peers. The odds were especially significant lower for those with epilepsy (OR 0.41, 95% CI: 0.38–0.44) (18 percentage points) and SMA, SB and CP (OR 0.23, 95% CI: 0.21–0.26) (31 percentage points) compared to healthy peers. Moreover, the results show that being a boy with an immigrant background and having parents with low SES reduces the odds of completing upper secondary education. Receiving mental health care treatment also reduces the odds of completing upper secondary education.

**Table 2:** The impact of long-term health challenges on the probability of school completion by age 21. Presented as OR (95% Cl) and average marginal effects. The sample is from birth cohorts in 1990 to 1996, *n* = 421,110

	OR	AME		
	OR (95% CI)	Coeff.	St.error	***P***-value
**Long-term health challenges** (ref: peers without long-term health challenges)				
Celiac disease	1.02 (0.94–1.11)	0.004	0.007	0.546
Asthma	1.04 (1.00–1.09)	0.007	0.004	0.048
IBD	0.87 (0.78–0.96)	− 0.026	0.010	0.009
Epilepsy	0.41 (0.38–0.44)	−0.185	0.007	0.000
Diabetes	0.80 (0.74–0.87)	− 0.043	0.008	0.000
Juvenile arthritis	0.88 (0.76–1.01)	−0.024	0.014	0.094
Sensory impairment	0.63 (0.59–0.67)	−0.090	0.006	0.000
SMA/SB/CP	0.23 (0.21–0.26)	−.0.313	0.014	0.000
**Gender** (ref:boys)				
Girls	1.62 (1.59–1.64)	0.087	0.001	0.000
**Immigrant background** (ref:majority)				
1.generaton	0.50 (0.49–0.52)	−0.014	0.003	0.000
2.generation	0.91 (0.87–0.94)	−0.180	0.003	0.000
**Parental education** (ref: Upper secondary education and below)				
Any college/university education	2.46 (2.43–2.50)	0.161	0.001	0.000
Unknown education	0.78 (0.74–0.82)	−0.056	0.006	0.000
**Parental income (log income)**	1.24 (1.22–1.27)	0.040	0.002	0.000
**Treated in mental health care**	0.26 (0.24–0.28)	−0.243	0.008	0.000
Constant	0.101 (0.08–0.13)			

Note: adjusted for birth cohorts

**Table 3:** The impact of long-term health challenges on the probability of NEET status at age 21. Presented as OR (95% Cl) and average marginal effects. The sample is from birth cohorts in 1990 to 1996, *n* = 421,110

	OR	AME		
	OR (95% CI)	Coeff.	St.error	***P***-value
**Long-term health** **challenges** (ref: peers without long-term health challenges)				
Celiac disease	1.15 (1.04–1.28)	0.014	0.006	0.012
Asthma	1.06 (1.00–1.11)	0.005	0.003	0.048
IBD	1.33 (1.17–1.51)	0.030	0.008	0.000
Epilepsy	3.41 (3.18–3.65)	0.180	0.007	0.000
Diabetes	1.53 (1.39–1.68)	0.048	0.006	0.000
Juvenile arthritis	1.67 (1.41–1.98)	0.060	0.012	0.000
Sensory impairment	1.78 (1.65–1.90)	0.068	0.005	0.000
SMA/SB/CP	9.14 (8.14–10.28)	0.403	0.014	0.000
**Gender** (ref:boys)				
Girls	0.77 (0.75–0.78)	−0.026	0.000	0.000
**Immigrant background** (ref:majority)				
1.generaton	1.67 (1.61–1.74)	0.060	0.003	0.000
2.generation	1.31 (1.25–1.37)	0.029	0.003	0.000
**Parental education** (ref: Upper secondary education and below)				
Any college/university education	0.60 (0.59–0.61)	− 0.049	0.000	0.000
Unknown education	1.43 (1.35–1.52)	0.047	0.004	0.000
**Parental income (log income)**	0.81 (0.80–0.83)	− 0.020	0.000	0.000
**Treated in mental health care**	2.80 (2.55–3.06)	0.101	0.004	0.000
Constant	1.932 (1.589–2.349)			

Note: adjusted for birth cohorts

**Table 4:** The impact of long-term health challenges on the probability of receiving disability pension by age 21. Presented as OR (95% Cl) and average marginal effects. The sample is from birth cohorts in 1990 to 1996, *n* = 421,110

	OR	AME		
	OR (95% CI)	Coeff.	St.error	***P***-value
**Long-term health challenges** (ref: peers without long-term health challenges)				
Celiac disease	1.67 (1.29–2.17)	0.007	0.002	0.002
Asthma	1.00 (0.84–1.18)	−0.000	0.000	0.977
IBD	1.39 (0.95–2.04)	0.004	0.003	0.141
Epilepsy	18.14 (16.58–19.85)	0.147	0.006	0.000
Diabetes	1.92 (1.49–2.48)	0.009	0.002	0.000
Juvenile arthritis	1.88 (1.19–2.96)	0.008	0.004	0.041
Sensory impairment	6.18 (5.50–6.95)	0.500	0.003	0.000
SMA/SB/CP	94.00 (83.70–105.59)	0.476	0.014	0.000
**Gender** (ref:boys)				
Girls	0.97 (0.92–1.02)	−0.000	0.000	0.334
**Immigrant background** (ref:majority)				
1.generaton	1.27 (1.13–1.42)	0.003	0.000	0.000
2.generation	1.21 (1.07–1.37)	0.002	0.000	0.005
**Parental education** (ref: Upper secondary education and below)				
Any college/university education	0.90 (0.85–0.95)	−0.001	0.000	0.000
Unknown education	1.67 (1.43–1.70)	0.009	0.002	0.000
**Parental income (log income)**	0.88 (0.86–0.90)	−0.002	0.000	0.000
**Treated in mental health care**	1.44 (1.23–1.70)	0.005	0.001	0.000
Constant	0.516 (0.040–0.067)			

Note: adjusted for birth cohorts

As shown in Table 3, the odds of NEET status by age 21 were significantly higher among young adults with long-term health challenges compared to healthy peers. This was true regardless of the type of long-term health challenge, but the odds were higher for certain diagnoses. For instance, young adults with sensory impairment had 1.78 times higher odds (0.7 percentage points), young adults with epilepsy 3.41 times higher odds (18 percentage points) and young adults with SMA, SB and CP had 9.14 times higher odds (40 percentage points) of NEET status compared to healthy peers. The results show that being a boy with an immigrant background and having parents with low SES increase the odds of having a NEET status by age 21. Receiving mental health care treatment also increases the odds of having a NEET status by age 21.

The results show that 41% of young adults with a long-term health challenge who had not completed upper secondary school also had a NEET status by age 21 (results not shown).

Young adults growing up with long-term health challenges had significantly higher odds of receiving disability pension by age 21 compared to healthy peers, except for young adults growing up with asthma and IBD (Table 4). As for the odds of receiving disability pension by age 21, the results show that young adults with epilepsy, sensory impairment and young adults with SMA, SB and CP have the highest odds of receiving disability pension compared to healthy peers. The results are especially striking in relation to young people with the long-term health challenges SMA, SB and CP who have 94 times higher odds (48 percentage points) of receiving disability pension by age 21 compared to healthy peers. Being treated in mental health care also increases the odds of receiving disability pension by age 21. The results show that 24% of young people with long-term health challenges with a NEET status by age 21 also received disability pension (results not shown).

### The impact of parent’s education on the odds of school completion, NEET status, and receiving disability pension by age 21, interaction terms between parental education and long-term health challenges

To estimate whether the impact of parental education on the outcome variables is dependent on the type of long-term health challenge, we have included an interaction term between parental education and long-term health challenges (Table 5). The results in Table 5 show that having parents with higher education increases the odds of completing upper secondary education and decreases the odds of NEET status by age 21 within all groups, including the reference group. In relation to disability pension, Table five shows that having parents with higher education decreases the odds of receiving disability pension by age 21 for the long-term health challenges SMA, SB and CP.

**Table 5:** The impact of parent’s education on the probability of school completion, NEET status, and receiving disability pension by age 21, interaction terms between parental education and long-term health challenges. Presented as differences in marginal effects between youth holding parents with any college/university education vs parents holding upper secondary education and below in different diagnosis groups

	School completion		Neet status		Disability pension	
	Diff.	***P***-value	Diff.	***P***-value	Diff.	***P***-value
Healthy peers - Any college/university education vs. upper secondary education and below	0.161	0.000	−0.047	0.000	−0.001	0.056
Celiac disease - Any college/university education vs. upper secondary education and below	0.163	0.000	−0.060	0.000	−0.006	0.158
Asthma - Any college/university education vs. upper secondary education	0.155	0.000	−0.053	0.000	−0.002	0.137
IBD - Any college/university education vs. upper secondary education and below	0.174	0.000	−0.067	0.000	−0.003	0.549
Epilepsy - Any college/university education vs. upper secondary education	0.174*	0.000	−0.089	0.000	−0.018	0.088
Diabetes - Any college/university education vs. upper secondary education	0.173	0.000	−0.042	0.001	−0.011*	0.018
Juvenile arthritis - Any college/university education vs. upper secondary education	0.121	0.000	−0.052	0.023	−0.013	0.095
Sensory impairment - Any college/university education vs. upper secondary education	0.157*	0.000	−0.071	0.000	−0.003	0.673
SMA/SB/CP - Any college/university education vs. upper secondary education	0.093*	0.001	−0.109	0.000	−0.110*	0.000

Note: * indicates when parental education influences the outcomes variables significantly different for the diagnosis groups compared to the reference groups

Moreover, Table five also shows how parental education influences the outcome variables differently for young adults with long-term health challenges compared to the reference group. The results showed that parental education had significantly less impact for the diagnoses epilepsy, sensory impairment and SMA, SB and CP compared to the reference group in relation to upper secondary school completion. Similar results were found for the diagnoses diabetes and SMA, SB and CP in relation to receiving disability pension by age 21.

## Discussion

The aim of this study has been to examine the educational and employment outcomes of growing up with long-term health challenges during adolescence. In this longitudinal study, we found that growing up with long-term health challenges greatly influences a young person’s educational and vocational outcomes in the transition to early adulthood. Young adults with long-term health challenges, except for the diagnoses celiac disease, asthma and juvenile arthritis were less likely to complete upper secondary education compared to young adults without long-term health challenges. A possible explanation for the variation between the different long-term health diagnoses might be the extent of burden of disease. Greater disease severity and stronger treatment side effects have been linked to poorer school experiences and outcomes [9]. Asthma for instance is a condition many adolescents “have grown out of” by the time they reach adulthood [36]. Nevertheless, our finding that most of the different types of long-term health challenges negatively impact upper secondary school completion is consistent with prior studies. For instance, McKinley Yoder and Cantrell [37] found in their recent systematic review that childhood-onset long-term health challenges were significantly associated with not completing upper secondary education within 4 years of enrolment. Previous research has shown that young people with long-term health challenges experience frequent school disruptions; such as poorer school attendance [38, 39], difficulties with concentration and higher rates of grade repetition [37, 40], often related to complex management of their long-term health challenge. Our results showed that adolescents with long-term health challenges such as SMA, SB and CP in particular had lower odds of completing upper secondary education compared to healthy peers. These individuals might experience more severe physical impairment, for instance they may have various developmental deficits in motor, cognitive, intellectual, communication and social functioning that can affect learning acquisition and participation in school [41]. This is supported by the study conducted by Sentenac, Lach [32] that shows that children with neurodisabilities, compared to those without, were less likely to have completed high school and less likely to have been enrolled in post-secondary education by age 25. Moreover, previous research shows that comorbid disease is high in children with epilepsy [42] and CP [43], and this may be one reason for why these groups stand out negatively on the outcome variables. In this study, the odds of NEET status in early adulthood were significantly higher among young adults with long-term health challenges compared to the reference population. Nearly half (41%) of those with a long-term health challenge who had not completed upper secondary school also had a NEET status by age 21. Several studies have shown that greater educational attainment is significantly related to being employed [15, 44, 45]. Completing upper secondary education can provide skills and knowledge, which can encourage continued participation in education and entry into the labour market. Findings based on a systematic review by Achterberg, Wind [14], support our results that factors such as motor impairment and epilepsy are related to negative employment outcomes in early adulthood. The jobs available to young people are more likely to involve physical job demands that may not be compatible with restrictive motor illnesses such as SMA, SB and CP. Furthermore, a lack of formal qualifications that could otherwise make one eligible for physically less demanding jobs may enforce labour market disadvantage in this group. Although epilepsy is not a physically visible condition and does not limit physical abilities, a metasynthesis by Chong, Jamieson [46] found that many adolescents growing up with epilepsy experience self-imposed restrictions such as fear of having seizures and associated risks of injury that greatly impacted their everyday life. This may also explain our finding that adolescents with epilepsy had been treated most in mental health care. In addition, they may experience decrements in cognitive function associated with having seizures and taking antiepileptic drugs, which can cause formal sanctions that limit driving status and work possibilities [47, 48]. Our findings showed that young adults with epilepsy, sensory impairment and SMA, SB and CP in particular, compared to healthy peers, have higher odds of receiving disability pension by age 21. Few studies have been conducted to date on receiving disability pension at an early age. However, a previous Norwegian prospective longitudinal study showed that young adults aged 18–26 received some sort of long-term social welfare benefits due to self-reported health complaints in adolescence [23]. Adolescents growing up with long-term health challenges such as SMA and CP are often affected by impaired physical abilities, such as spasticity, dyskinesia and ataxia [49]. Adolescents with SB may have bladder and bowel dysfunction [50]. Furthermore, adolescents with such neurological conditions experience musculoskeletal pain, which has been found to be an important predictor of subsequent sickness and social welfare benefit receipt from adolescence to young adulthood [24]. The second aim of this article was to investigate the effects of parental education on the three outcome variables within the specific types of long-term health challenges. Previous research has shown that young people from more educated families, especially when parents have a university degree, are more likely to access education and employment after upper secondary school [32]. This is in line with the results in the present study. In relation to the discrepancies between the different diagnoses, our results showed that parental educational attainment had a lower impact on completing upper secondary school among adolescents with epilepsy, sensory impairment and SMA, SB and CP compared to adolescents without health impairments in this study. Parental education attainment also had a lower impact on receiving disability pension among young adults with diabetes and SMA, SB and CP compared to adolescents without health impairments by age 21. A possible explanation for this might be that young people living with these diagnoses have severe long-term health challenges that, despite their parents’ resources, still cause profound difficulties in their everyday lives.

### Strengths and limitations

One strength of this study is its longitudinal design, which allowed us to examine associations between long-term health challenges and upper secondary school completion, NEET status and disability pension in the transition from adolescence to young adulthood in a large study sample (*n* = 421,110). Moreover, the data used in this study were objective measures, ruling out any reporting bias. Another strength of this study is the inclusion of data on adolescents with different types of long-term health challenges, which made it possible to analyse variations in outcomes between diagnosis groups. However, one limitation is that some of the adolescents included in this study may have various combinations and degree of co-morbidities. The diagnoses recorded in NPR have been found to be sensitive to factors such as different coding and diagnostic practice, especially in terms of co-morbidities [51]. Underreporting of secondary diagnosis is expected [52]. Therefore, in conducting the analysis, the main diagnosis which the adolescents were registered with in the NPR registry was chosen, as done in other studies [53]. Children with comorbid physical illness are likely to present with greater emotional and social impairment than children without such comorbidities [54, 55]. However, the literature is scarce, and thus more knowledge of the prevalence and impact of medical comorbidity is needed and should be the subject in future studies. Furthermore, the reference population will include a certain, but unknown proportion of unhealthy peers. Thus, the statistical effects of ill health on the outcomes in the study population are conservative estimates. In observational analysis residual confounding is always a possibility and thus a limitation to drawing causal inferences. This said, we have made serious attempts to reduce this possibility by adding many, but of course not all, variables that are likely to influence the outcomes of interest. Further, logistic regression in particular may yield biased estimates because, unlike linear regression, the effect estimates are influenced by omitted variables, even when they are unrelated to the independent variables in the equation [34]. One way to remedy this problem is to calculate average marginal effects as we have done in this article. In sum, we have implemented several measures to deal with the problem of residual confounding. Nevertheless, any claim of causality between exposures, i.e. somatic diseases, and the three social outcomes should be made with utmost caution. NPR includes information on patients in contact with the specialist health services. Thus, the diagnoses used in this work are those recorded in NPR by medical specialists. The making of these diagnoses are thus in principle sensitive to a variety of factors like differences in access to and use of health services, different coding and diagnostic practice, and variation in symptoms presented to a physician. Evidence does indicate, however, that severe diseases recorded in NPR are valid: A comparison of cancer diagnoses recorded in NPR with the equivalent diagnoses recorded in the Cancer Registry showed a high degree of agreement [56]. Since the somatic diseases we have selected for scrutiny in this article also are severe, there is reason to assume that their validity is acceptable. Another limitation is that NEET status was only measured by age 21. The NEET group includes several sub-groups, including those who have full control of their life situation and are not looking for work or applying for education and are not constrained to do so because of limitations or incapacities, and those who are engaged in activities such as art and travelling [57]. Another limitation is that we did not have any information in the dataset on the severity of the different health conditions included, which most likely affects the odds of completing upper secondary school, NEET status and receiving disability pension.

### Implications and conclusion

Young adults growing up with long-term health challenges have greater odds of not completing upper secondary education 5 years after enrolment, NEET status and receiving disability pension by age 21 compared to those without a long-term health challenge. The difference is particularly striking among young adults who have grown up with neurological conditions such as SMA, SB and CP. This indicates a process of cumulative disadvantage, in which young people with reduced motor functioning struggle to access and participate in education and employment. This implicates that policy-makers and professionals within the health, educational and social system should be aware that especially these young people need adequate support or training transitioning into adulthood. Furthermore, it appears as if for some young people, the more severe the long-term health challenge is, despite their parents’ resources they still have difficulties with participating in education and employment. This suggests that parents need assistance in coping and adapting to the complex situation as well. Active collaboration among the young people, parents and service providers may contribute to facilitate a better transition from school to work. By understanding the barriers and needs from the young people and parent’s perspective, the shortcomings of the current policy systems can be identified and strategies to improve services can be developed. This should be followed up in future studies. Additionally, future studies may examine the availability and utilization of work accommodations and benefits for young people with long-term health challenges at the early career phase.

## Data Availability

The data that support the findings of this study are available from Statistic Norway, but restrictions apply to the availability of these data, which were used under licence for the current study, and so are not publicly available. Data are however available from the authors upon reasonable request and with permission of Statistic Norway.

## Appendix

**Table 6:** Supplementary information about the different long-term health challenges

Asthma	The prevalence of asthma various with how asthma is defined and which methods are used to measure it. Figures from Scandinavia shows that the prevalence is increasing, between 4 -10% of children under the age of 15 has asthma [58].
Epilepsy	The prevalence of epilepsy among children in the Nordic countries varies from 3.2–5.1 cases per 1000 persons [59].
Diabetes type 1	The incidence of type 1 diabetes in Norway is 28/100,000 per year in children below the age of 15 [60].
Sensory impairment (hearing and visual impairment)	Permanent hearing impairment is estimated to occur in 1–2 children per 1000 newborns in developed countries [61, 62]. The prevalence of visual impairment in children and adolescents in the Nordic countries are quite similar [63], with prevalence rates of 11 per 10,000 (0–19 years) [64].
Celiac disease	Celiak disease is a long-term inflammation in the proximal small intestine caused by an inappropriate immune response against gluten proteins contained in wheat, barley, and rye. Celiak disease is increasing in Norway among children, with the incidence rate 45.5 cases per 100,000 person-years below the age of 15 [65].
Inflammatory bowel disease (IBD)	IBD is a term for two conditions (chrohn’s disease and ulcerative colitis) that are characterized by long-term inflammation of the gastrointestinal (GI) tract. Incidence rates for pediatric chrons disease have increased up to 9 or 10 per 100,000 population in parts of Europe, including Scandinavia, while rates for pediatric ulcerative colitis are often slightly lower than for chrons disease [66].
Juvenile arthritis	Juvenile arthritis is a disease that causes inflammation in one or more joints (arthritis), and where the disease starts before the age of 16. The reported prevalence is around 1–2 cases per 1000 children [67, 68].
Spinal muscular atrophy (SMA)	SMA is characterised by degeneration of the alpha motor neurons of the spinal cord anterior horn cells, leading to progressive proximal muscle weakness and atrophy. Prevalence of approximately 1–2 per 100,000 persons have been estimated with SMA [69].
Spina bifida (SB)	SB is a congenital birth defect that is caused by a failed closure of one or more vertebrae during the early weeks of gestation. Every year, 20–30 children are born with spina bifida in Norway. This corresponds to 4–5 cases per 10,000 births [70].
Cerebral palsy (CP)	CP is the most common cause of permanent motor disabilities in children [71]. The average prevalence of CP for children born in Norway 1999 to 2010 was 2.35 per 1000 live births [72].

## Abbreviations

SMA: Spinal muscular atrophy
SB: Spina bifida
CP: Cerebral palsy
FD-Trygd: Historical event database
ICD-10: International statistical classification of diseases and related health problems
NUDB: National Education Database
NPR: Norwegian Patient Registry
NEET status: Not in education, employment or training
SSB: Statistics Norway
*p* value: Probability value
SD: Standard deviations
SE: Standard error
